# Decreased mortality in acute respiratory distress syndrome patients treated with corticosteroids: an updated meta-analysis of randomized clinical trials with trial sequential analysis

**DOI:** 10.1186/s13054-021-03546-0

**Published:** 2021-03-26

**Authors:** Ping Lin, Yuean Zhao, Xiaoqian Li, Faming Jiang, Zongan Liang

**Affiliations:** Department of Respiratory and Critical Care Medicine, West China School of Medicine and West China Hospital, Sichuan University, Chengdu, 610041 Sichuan China

**Keywords:** Acute respiratory distress syndrome, Glucocorticoids, Randomized clinical trial, Meta-analysis

## Abstract

**Background:**

The possible benefits associated with corticosteroid treatment in acute respiratory distress syndrome (ARDS) patients are not fully known. We conducted an updated meta-analysis to assess the effect of corticosteroids in the treatment of patients with ARDS.

**Methods:**

We systematically searched MEDLINE, Embase, and the Cochrane Library from inception to January 2021 via Ovid to identify randomized controlled trials evaluating the efficacy of glucocorticoids in the treatment of patients with ARDS. The primary outcome was hospital mortality. Secondary outcomes included the number of ventilator-free days at day 28, oxygenation improvement (PaO_2_/FIO_2_ ratios), and adverse events.

**Results:**

Nine studies with 1371 participants were analyzed. The pooled analysis revealed that glucocorticoid use was associated with reduced mortality [relative risk (RR), 0.83; 95% confidence interval (CI) 0.74–0.93; *P* < 0.01; *I*^2^ = 37], and the statistical power was confirmed by trial sequential analysis. Glucocorticoids might also significantly increase the number of ventilator-free days at day 28 (mean deviation 3.66 days, 95% CI 2.64–4.68; *P* < 0.01) and improve oxygenation (standardized mean difference 4.17; 95% CI 2.32–6.02; *P* < 0.01). In addition, glucocorticoid use was not associated with increased risks of new infection (RR 0.84; 95% CI 0.70–1.01; *P* = 0.07) and hyperglycemia (RR 1.11; 95% CI 0.99–1.23; *P* = 0.06).

**Conclusions:**

The use of glucocorticoids might result in reduced mortality in patients with ARDS. Glucocorticoids might be recommended as an adjunct to standard care for ARDS; however, the optimal dose and duration of steroid therapy remains unknown and further studies are needed.

**Supplementary Information:**

The online version contains supplementary material available at 10.1186/s13054-021-03546-0.

## Introduction

The acute respiratory distress syndrome (ARDS) is a life-threatening condition characterized by bilateral pulmonary infiltrates on chest imaging and refractory hypoxemia [1]. It is not uncommon in critically ill patients in intensive care units (ICUs) and is associated with considerable mortality [2]. A recent international study found that nearly 10% of ICU patients suffered from ARDS, and the hospital mortality of ARDS patients was about 40% [3]. Despite decades of research, current pharmacological therapies for ARDS are limited [4]. Overwhelming lung inflammation plays a key role in the pathogenesis of ARDS [5]. Therefore, inflammation-directed therapies, such as glucocorticoid treatment, appear to be a reasonable strategy to treat ARDS patients.

Glucocorticoids have anti-inflammatory and anti-fibrosis effects and have been the most investigated immunomodulatory agent for the treatment of ARDS. However, the impact of corticosteroid therapy on clinically relevant outcomes in ARDS patients remains controversial. Clinical trials evaluating corticosteroids in the management of ARDS reported conflicting results [6–8], and previous meta-analyses were underpowered to draw determinate conclusions [9, 10]. Thus, we conducted an updated meta-analysis with trial sequential analysis to assess the effect of corticosteroids in the treatment of patients with ARDS and determine whether the current evidence is reliable.

## Methods

### Data sources and searches

This meta-analysis was conducted according to the Preferred Reporting Items for Systematic Reviews and Meta-Analyses statement (PRISMA) [11]. We systematically searched MEDLINE, Embase, and the Cochrane Library from inception to January 2021 via Ovid to identify randomized controlled trials (RCTs), using the following search terms: (“ALI” OR “acute lung injury” OR “ARDS” OR “acute respiratory distress syndrome”) AND (“steroids” OR “corticoid” OR “corticosteroid” OR “glucocorticoids” OR “hydrocortisone” OR “prednisolone” OR “dexamethasone” OR “methylprednisolone”) AND (“randomized controlled trial” OR “controlled clinical trial” OR “randomized” OR “randomly” OR “trial”). The detail of the search strategy is shown in Additional file 1. Publication species were limited to humans. Besides, bibliographies of identified studies were also searched manually.

### Study selection

The eligibility of each study identified from the literature search was assessed independently in a blinded fashion by two researchers. English-language, peer-reviewed studies meeting the following criteria were included in this meta-analysis: (1) study design: RCTs; (2) participant: adult patients with ARDS; (3) intervention: glucocorticoids versus control; (4) at least one of the following outcomes: all-cause mortality, number of ventilator-free days at day 28, oxygenation improvement (PaO_2_/FIO_2_ ratios), and adverse events. Any discrepancies were resolved through discussion.

### Data abstraction and quality assessment

Using a standardized data collection form, two investigators independently extracted the following data from each eligible study: first author, publication year, number of patients, inclusion criteria, corticosteroid type, corticosteroid dose, therapy duration, and main outcomes. The primary outcome was hospital mortality. If hospital mortality was not reported, we used the closest time point for our analysis. Secondary outcomes included the number of ventilator-free days at day 28, PaO_2_/FIO_2_ ratios, and adverse events.

The Cochrane risk-of-bias tool was used to assess the risk of bias of the eligible studies [12]. This tool comprises seven domains, and each domain scores as low, unclear, or high risk of bias: adequate sequence generation, allocation concealment, blinding of participants and personnel to the research protocol, blinding of outcome assessment, incomplete outcome data, selective reporting, and other bias. Any discrepancies were resolved through discussion.

### Data synthesis

Risk ratios (RRs) with 95% confidence intervals (CIs) were calculated for dichotomous outcomes. Weighted mean differences (MDs) or standardized mean differences (SMDs) with their 95% CIs were calculated for continuous data. If only medians and interquartile ranges were available, means and standard deviations (SDs) were estimated according to the methods described by Hozo et al. [13]. Heterogeneity across eligible studies was assessed using the *I*^2^ statistic. There was significant heterogeneity if the *I*^2^ value > 50% [14]. We analyzed all data using fixed-effects models if the *I*^2^ value < 50%; otherwise, random-effects models were used. The risk of publication bias was assessed by a funnel plot. All *P* values were two-sided, and a *P* value < 0.05 was recognized as statistically significant. Review Manager Software (version 5.4, The Cochrane Collaboration) was used for all statistical analysis.

Trial sequential analysis (TSA), a method that can correct for the increased risk of type I errors caused by sparse data and repeated significance testing on accumulating data and can determine whether the evidence in a meta-analysis is reliable, was conducted in our study [15, 16]. When the cumulative *Z* curve crosses the futility boundary or the trial sequential monitoring boundary, there is sufficient evidence to reach a conclusion and no further trials are needed to confirm the results. We conducted the trial sequential analysis to estimate the required information size using a type I error of 5%, a type II error of 20% (power 80%), an anticipated relative risk reduction of 20%, and the control event proportions were calculated from the control group. We used TSA version 0.9.5.10 beta (www.ctu.dk/tsa) for the analyses.

## Results

### Search results

**Fig. 1:**
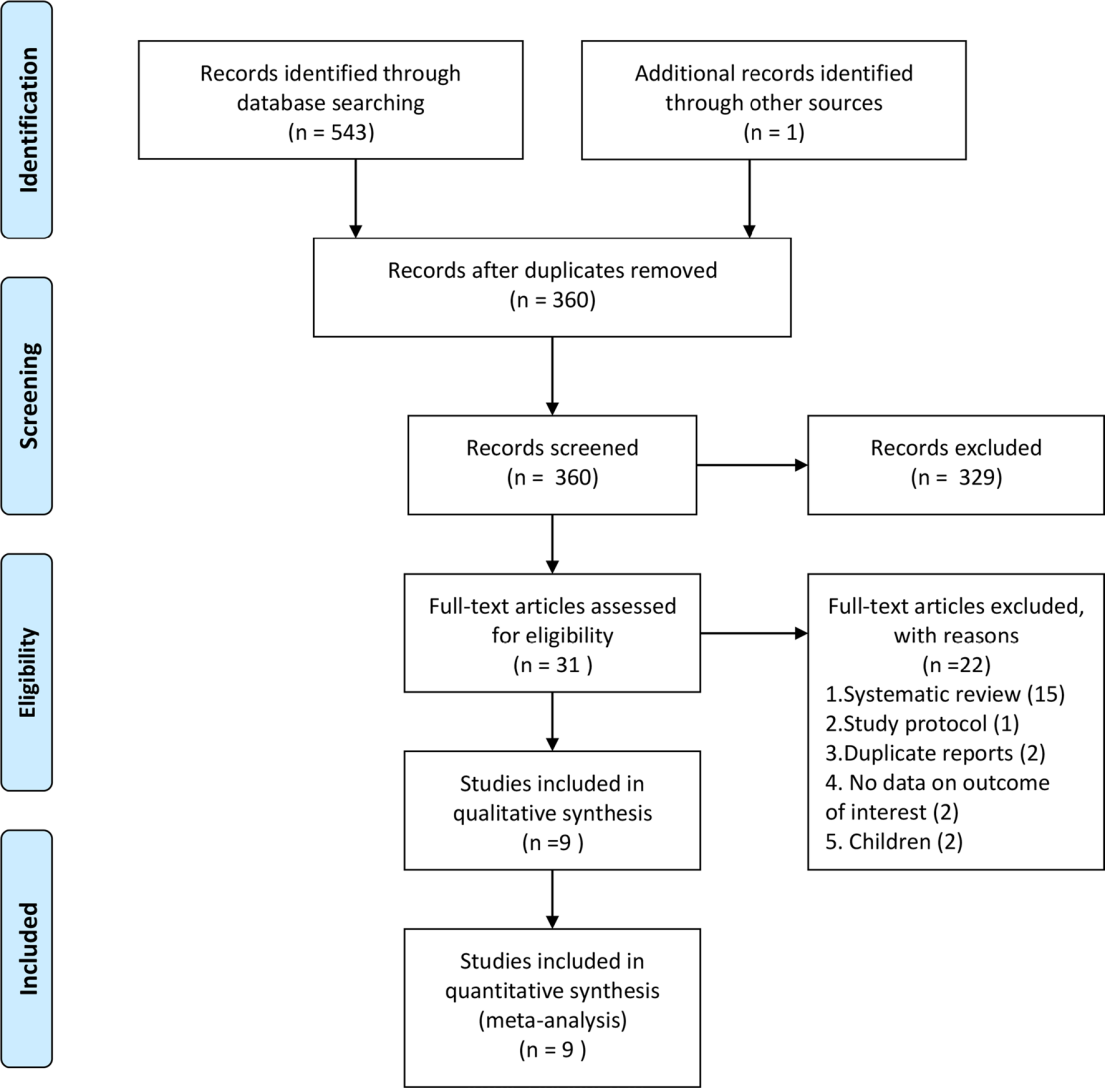
PRISMA 2009 flow diagram of identified studies

A total of 544 potentially eligible records were identified by a comprehensive literature search. After excluding duplicates and checking the titles and abstracts, thirty-one studies were retrieved. After reviewing the full text, nine studies met all eligibility criteria and were included in the current meta-analysis [17–25]. The detailed flowchart for literature selection is shown in Fig. 1.

### Characteristics of studies

The main characteristics of the eligible studies are summarized in Table 1. These studies were published between 1987 and 2020. The number of included participants from each study ranged from 24 to 299 (total 1371). All participants met moderate-to-severe ARDS criteria (PaO_2_/FIO_2_ ≤ 200). Corticosteroid regimens varied apparently among studies. Of these, five studies used methylprednisolone, two hydrocortisone, and two dexamethasone. Treatment duration ranged from 1 to 28 days. Corticosteroid dose was also different among studies, ranging from 1 to 120 mg/kg/d of methylprednisolone or equivalent.

**Table 1:** The main characteristics of the randomized controlled trials

Study	No. of patients (steroids/control)	Subjects	Intervention	Treatment duration	Main outcomes
Bernard/1987	50/49	(1) Patients with PaO_2_ ≤ 70 mmHg (FiO_2_ ≥ 40%) or PaO_2_/PAO_2_ ≤ 0.3; (2) bilateral diffuse infiltrates on chest radiography; (3) PAWP ≤ 18 mmHg	Methylprednisolone 30 mg/kg IV every 6 h	24 h	45-day all-cause mortality
Meduri/1998	16/8	(1) patients diagnosed with ARDS by the AECC definition; (2) 7 days of mechanical ventilation with an LIS ≥ 2.5 and less than 1-point reduction from day 1 of ARDS; (3) no evidence of untreated inflection	Methylprednisolone 2 mg/kg bolus followed by 2 mg/kg (day 1–14), 1 mg/kg (day 15–21), 0.5 mg/kg (day 22–28),0.25 mg/day (day 28–32)	32 days	Improvement in lung function and mortality
Annane/2006	85/92	(1) septic shock patients with bilateral infiltrates on chest radiography; (2) PaO_2_/FIO_2_ ≤ 200; (3) PAWP ≤ 18 mmHg or no clinical evidence of left atrial hypertension	Hydrocortisone 30 mg IV every 6 h and 9-fludrocortisone 50ug orally once a day	7 days	28-day survival
Steinberg/2006	89/91	(1) adult patients diagnosed with ARDS by the AECC definition; (2) patients were intubated and mechanically ventilated for 7–28 days after the onset of ARDS	Methylprednisolone 2 mg/kg bolus followed by 2 mg/kg (day 1–14), 1 mg/kg (day 15–21), tapering over (day 22–25)	25 days	60-day all-cause mortality
Meduri/2007	63/28	Adult intubated patients diagnosed with ARDS by the AECC definition	Methylprednisolone 1 mg/kg bolus followed by 1 mg/kg (day 1–14), 0.5 mg/kg (day 15–21), 0.125 mg/kg (day 22–25), 0.25 mg/day (day 26–28)	28 days	A 1-point reduction in LIS or successful extubation by day 7
Rezk/2013	18/9	(1) ARDS patients with PaO_2_/FIO_2_ < 200, bilateral pulmonary infiltrates, and PAWP < 18 mmHg; (2) patients were mechanically ventilated	Methylprednisolone 1 mg/kg bolus followed by 1 mg/kg (day 1–14), 0.5 mg/kg (day 15–21), 0.125 mg/kg (day 22–25), 0.25 mg/day (day 26–28)	28 days	Improvements of clinical parameters
Tongyoo/2016	98/99	(1) Adult patients with severe sepsis or septic shock receiving mechanical ventilation; (2) patients diagnosed with ARDS by the AECC definition and the Berlin criteria	Hydrocortisone 50 mg IV every 6 h	7 days	28-day all-cause mortality
Villar/2020	139/138	(1) Adult patients were intubated and mechanically ventilated; (2) patients diagnosed with ARDS by the AECC definition or the Berlin criteria as moderate to severe ARDS	Dexamethasone 20 mg once daily from day 1 to day 5, which was reduced to 10 mg once daily from day 6 to day 10	10 days	Ventilator-free days during the first 28 days
Tomazini/2020	151/148	(1) Adult patients with confirmed or suspected COVID-19 infection were receiving mechanical ventilation; (2) patients diagnosed with moderate to severe ARDS by the Berlin criteria	Dexamethasone 20 mg daily from day 1 to day 5, followed by 10 mg daily for 5 days or until ICU discharge	10 days or until ICU discharge	Ventilator-free days during the first 28 days

*ARDS* acute respiratory distress syndrome, *PaO*_*2*_ partial pressure of oxygen in arterial blood, *PAO*_*2*_ partial pressure of alveolar oxygen, *FiO*_*2*_ fraction of inspired oxygen, *PAWP* pulmonary artery wedge pressure, *AECC* the American-European Consensus Conference criteria, *LIS* lung injury score, *COVID-19* coronavirus disease 2019, *ICU* intensive care unit, *IV* intravenous, *h* hour(s)

### Risk of bias

Table 2 illustrates the details of the risk of bias assessment. Overall, seven studies achieved a low overall risk of bias [17–21, 23, 24], and two studies were judged to be at high risk of bias [22, 25]. Among the eligible studies, eight studies generated an adequate randomization sequence and seven studies reported appropriate allocation concealment. Blinding was conducted in most trials except two studies [22, 25].

**Table 2:** Assessment of risk of bias using the Cochrane risk-of-bias tool

Study	Random sequence generation	Allocation concealment	Blinding of participants and personnel	Blinding of outcome assessment	Incomplete outcome data	Selective reporting	Other bias
Bernard/1987	Low	Low	Low	Low	Low	Unclear ^A^	Low
Meduri/1998	Low	Low	Low	Low	Low	Low	Low
Annane/2006	Low	Low	Low	Low	Low	Low	Low
Steinberg/2006	Low	Low	Low	Low	Low	Low	Low
Meduri/2007	Low	Low	Low	Low	Low	Low	Low
Rezk/2013	Unclear^A^	High	High	High	Unclear ^A^	Unclear ^A^	Unclear ^A^
Tongyoo/2016	Low	Low	Low	Low	Low	Low	Low
Villar/2020	Low	Low	Low	Low	Low	Low	Low
Tomazini/2020	Low	High	High	High	Low	Low	Low

^A^Indicating insufficient information

### Mortality outcomes

**Fig. 2:**
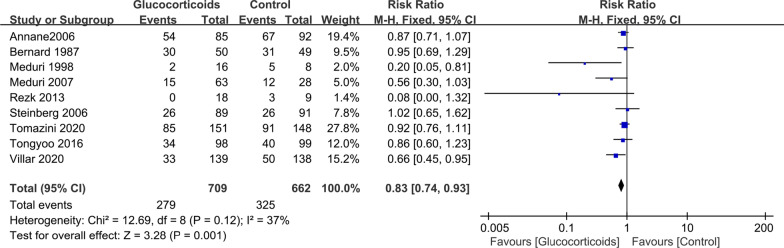
The effect of glucocorticoid treatment on mortality. *CI* confidence interval

**Fig. 3:**
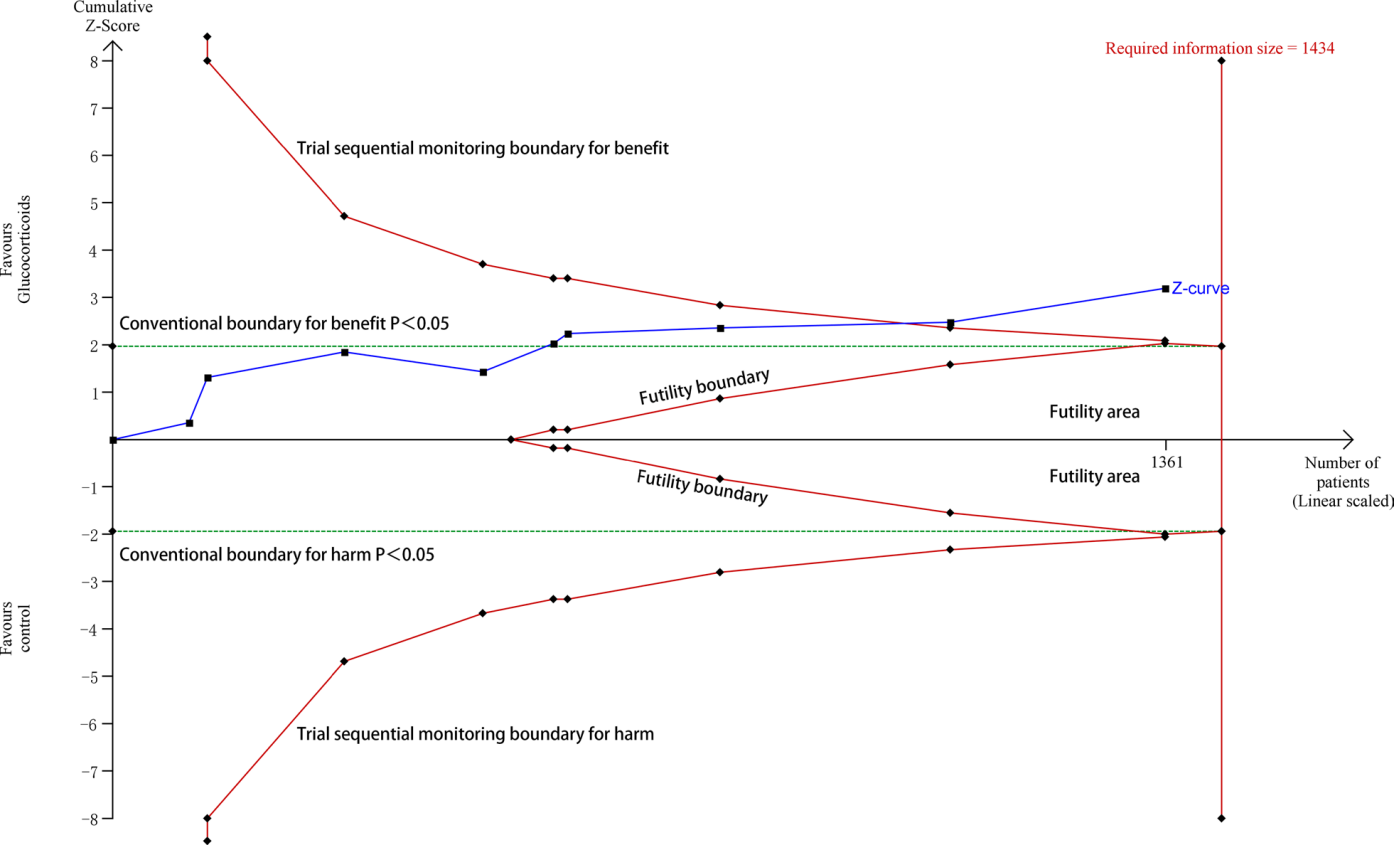
Trial sequential analysis of nine trials for hospital mortality. The required information size for detecting an intervention effect was 1434 patients. The relative risk was 0.83, and the 95% confidence interval was corrected to 0.74–0.94, from 0.74 to 0.93. The cumulative *Z* curve crossed both the conventional boundary for benefit and the trial sequential monitoring boundary for benefit

Between 1987 and 2020, nine studies with 1371 participants presented available results on mortality [17–25]. The mortality in the glucocorticoid group and the control group was 39.4% (279 of 709 patients) and 49.1% (325 of 662 patients), respectively. The pooled results showed that glucocorticoids are associated with reduced hospital mortality (RR 0.83; 95% CI 0.74–0.93; *P* < 0.01), with no significant heterogeneity (*I*^2^ = 37, *P* = 0.12) (Fig. 2). The TSA results showed that the adjusted 95% CI of RR was 0.74–0.94, and the required information size for detecting an intervention effect was 1434 patients. The cumulative *Z* curve crossed both the conventional boundary for benefit and the trial sequential monitoring boundary for benefit (Fig. 3), suggesting that current evidence is sufficient and further studies are unlikely to change the current conclusion of benefit with glucocorticoids. There was a potential risk of publication bias in favor of positive findings by inspection of the funnel plot (Additional file 2). After excluding two small studies with low weight [20, 25], glucocorticoids were still associated with reduced hospital mortality (RR 0.85; 95% CI 0.76–0.95; *P* = 0.006; *I*^2^ = 0%).

### Number of ventilator-free days at day 28

**Fig. 4:**
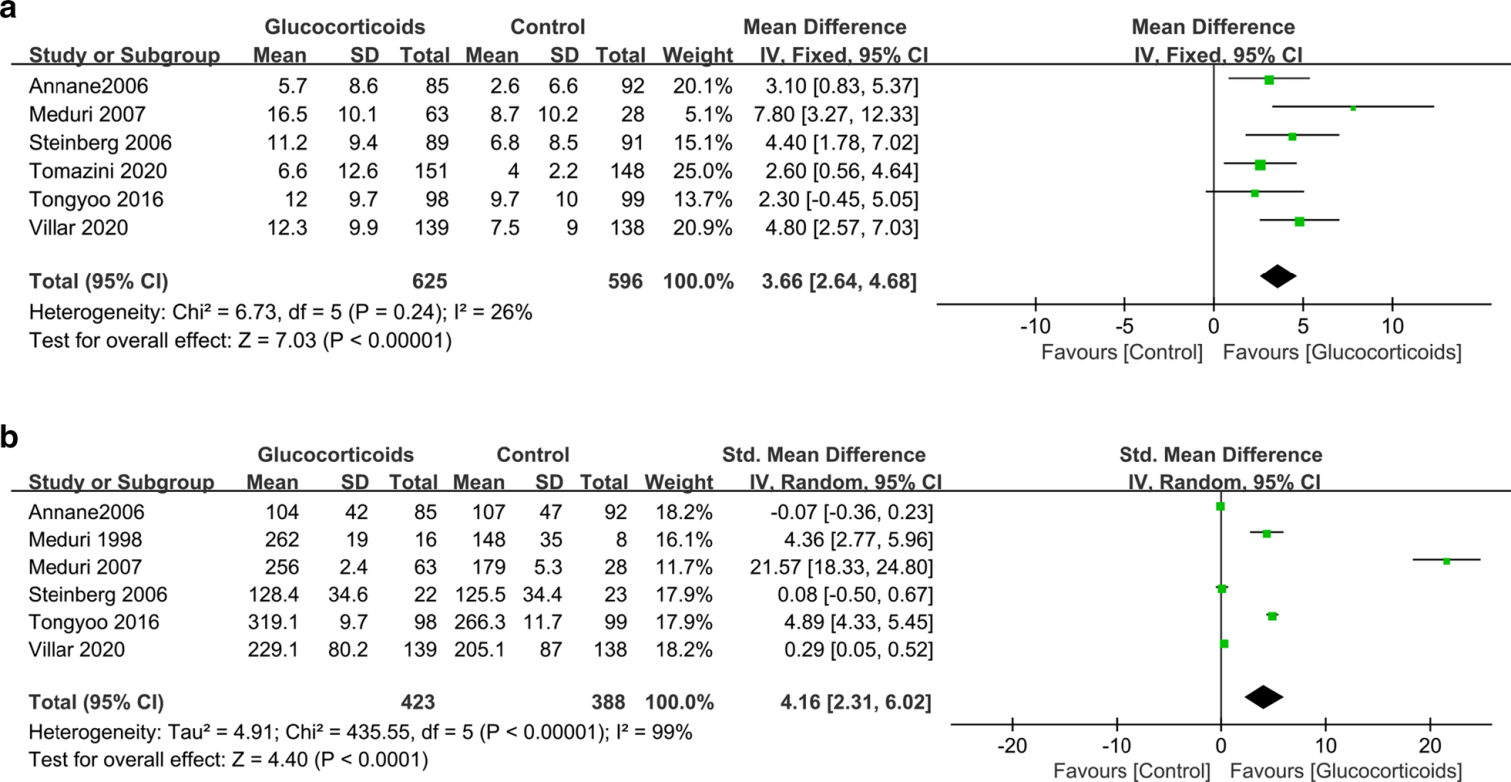
The effect of glucocorticoid treatment on the number of ventilator-free days at day 28 (**a**) and PaO_2_/FIO_2_ ratios (**b**). *CI* confidence interval

Six trials had data on the number of ventilator-free days at day 28 [17, 19, 21–24]. The pooled result showed that glucocorticoid use was associated with more ventilator-free days at day 28, with a mean difference of 3.66 days (95% CI 2.64–4.68; *P* < 0.01; *I*^2^ = 26%) (Fig. 4a).

### PaO_2_/FIO_2_ ratios

Six trials investigated oxygenation improvement (PaO_2_/FIO_2_ ratios) of the glucocorticoid versus control groups [17, 19–21, 23, 24]. The pooled result showed that glucocorticoid use might significantly improve oxygenation (SMD, 4.16; 95% CI 2.31–6.02; *P* < 0.01; *I*^2^ = 99%) (Fig. 4b).

### Adverse events

**Fig. 5:**
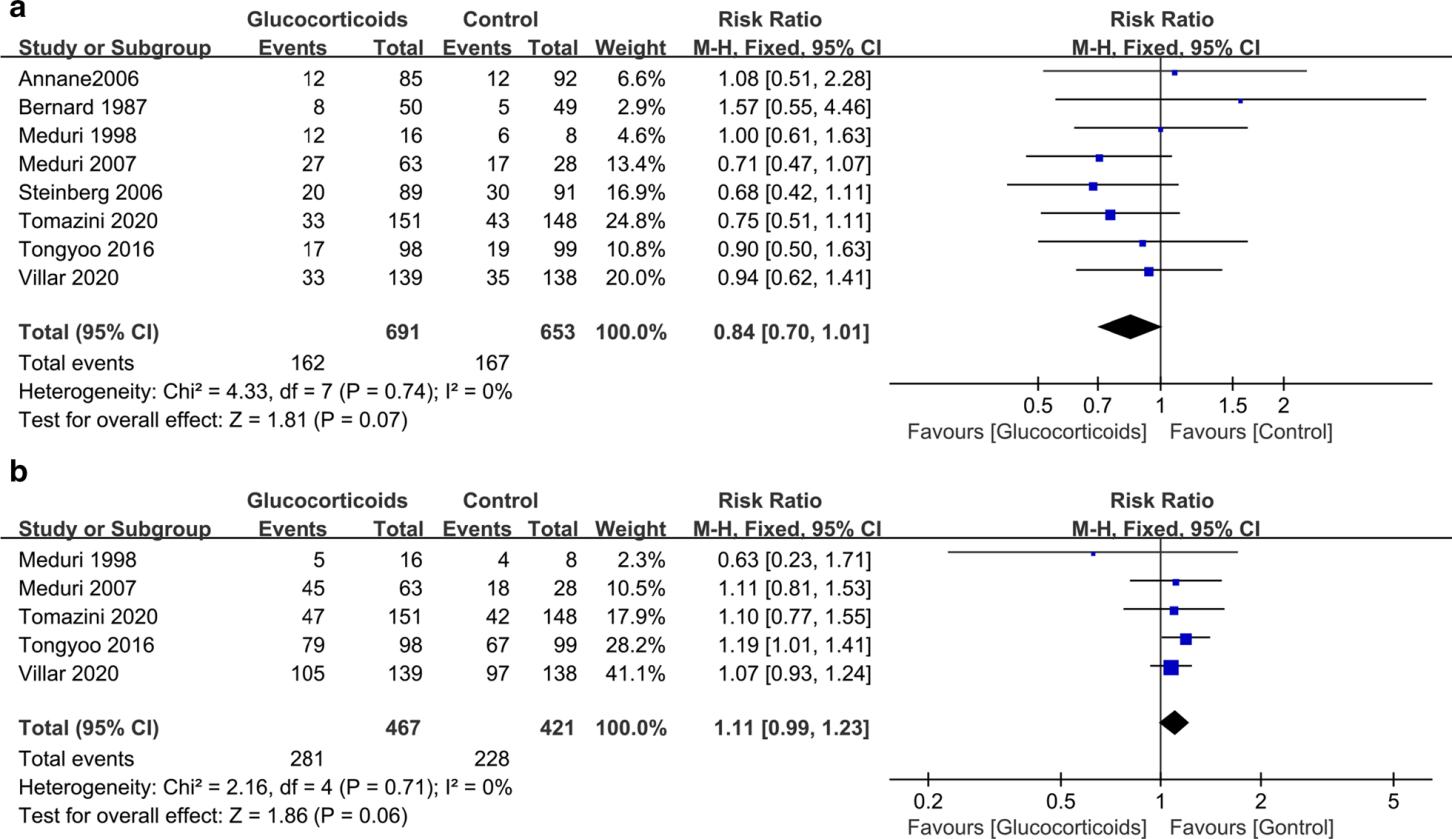
The effect of glucocorticoid treatment on new infection (**a**) and hyperglycemia (**b**). *CI* confidence interval

Data on new infection and hyperglycemia were available in eight studies [17–24] and five studies [19, 20, 22–24], respectively. The pooled result showed that glucocorticoid treatment was not associated with a higher incidence of new infection (RR 0.84; 95% CI 0.70–1.01; *P* = 0.07; *I*^2^ = 0) (Fig. 5a). TSA showed that the cumulative *Z*-curve did not crossed both the conventional boundary and the trial sequential monitoring boundary (Additional file 3), indicating that current evidence is inconclusive and further studies are needed. Glucocorticoid treatment was also not associated with an increased risk of hyperglycemia (RR 1.11; 95% CI 0.99–1.23; *P* = 0.06; *I*^2^ = 0) (Fig. 5b), which was confirmed by TSA (the cumulative *Z*-curve surpassed the futility boundary) (Additional file 4).

## Discussion

This was an updated meta-analysis of RCTs to evaluate the benefits and risks associated with glucocorticoid use in ARDS patients. In our meta-analysis, we found that glucocorticoids might reduce mortality and duration of mechanical ventilation and improve oxygenation in patients with ARDS. Besides, this study also indicated that glucocorticoid treatment was not associated with a higher incidence of new infection and hyperglycemia.

Several systematic reviews and meta-analyses on the topic had been presented [9, 26–29]. Among previous meta-analyses, Zayed, Y.'s study was the most comprehensive one [9]. It included eight RCTs totaling 1091 patients for analysis and found that glucocorticoid use was associated with a significant reduction in-hospital mortality (RR 0.79; 95% CI 0.64–0.98; *P* = 0.03). Unfortunately, TSA suggested insufficient information size and potentially false-positive results in Zayed, Y.'s study. Although the main outcomes of our meta-analysis were consistent with those reported in Zayed, Y.'s study, there were significant differences between our study and Zayed, Y.'s study. Firstly, our study did not include one RCT which was included in Zayed, Y.'s meta-analysis because this RCT recruited severe pneumonia patients instead of ARDS [30]. Secondly, the present study included two additional RCTs which were published recently, with an added statistical power of about 300 cases [22, 25]. Our study was the most comprehensive meta-analysis and reinforced the earlier results of previous meta-analyses. Thirdly, we used TSA to estimate the effect more conservatively in the present study. Finally, this was the first meta-analysis with sufficient evidence to confirm that glucocorticoids might significantly reduce mortality in patients with ARDS and further studies are unlikely to change the current conclusion.

There were some meta-analyses exploring the effects of corticosteroids on other conditions such as coronavirus disease 2019 (COVID-19) [31], sepsis [32], and community-acquired pneumonia [27]. Glucocorticoid use was found to be associated with significant improvement in clinical outcomes in these conditions. One possible reason for these findings was that critical illness might be associated with an impaired hypothalamic–pituitary–adrenal (HPA) axis response to stress [33]. ARDS is a devastating lung disorder and is associated with a high mortality rate. One study showed that critical illness-related corticosteroid insufficiency (CIRCI) was common in ARDS (about 58%) and stress dose glucocorticoid was associated with prolong survival time [34]. The early use of corticosteroid therapy might alleviate systemic inflammation caused by CIRCI and then was associated with survival benefit.

An anti-inflammatory pharmacologic intervention appears to be a reasonable strategy in ARDS in that dysregulated and excessive pulmonary inflammation is the pathophysiologic hallmarks of ARDS [35]. Among the anti-inflammatory drugs, glucocorticoids are the main immunomodulatory agent for the treatment of ARDS. Early studies demonstrated that glucocorticoid treatment led to rapid improvements in pulmonary and extrapulmonary organ function in ARDS patients, with a significant reduction in bronchoalveolar lavage and plasma levels of proinflammatory mediators and chemokines [36, 37]. Animal experiments also found that corticosteroid treatment could alleviate lung injury and upregulate pulmonary glucocorticoid receptors [38, 39]. Unfortunately, clinical trials reported inconsistent results. Some trials found that glucocorticoids might reduce the risk of death in patients with ARDS [20, 24]. However, there also were some trials that failed to find such benefits [19, 23]. Until today, glucocorticoid use in ARDS remains highly controversial due to unclear benefits and potential side effects [40]. Our meta-analysis pooled the latest data and suggested that glucocorticoids show beneficial effects in patients with ARDS without significant side effects. Therefore, glucocorticoids might be recommended as an adjunct to standard care for ARDS due to the beneficial effects. In addition, there has been significant progress in the management of ARDS in the past 20 years. High-quality clinical trials have confirmed that ventilation with lower tidal volumes and prone positioning can significantly decrease all-cause mortality of ARDS after 2000 [41, 42]. With the progress of standards of care for patients with ARDS, whether glucocorticoid use can reduce the mortality rate of ARDS should be noted. When limiting to trials commenced after 2000 [22–25], we found that glucocorticoid use is also associated with reduced mortality (RR 0.81; 95% CI 0.69–0.95; *P* = 0.01; *I*^2^ = 46), indicating glucocorticoid use is still applicable today.

One might expect that glucocorticoid use could cause adverse events such as new infections and hyperglycemia in patients with ARDS. However, we did not find significant differences between the glucocorticoid group and the control group in the incidence of adverse events. The use of low-dose glucocorticoids in the experimental group might be responsible for the unexpected outcomes. Among eligible studies, except for one trial that used high-dose glucocorticoids (methylprednisolone 30 mg/kg every 6 h) for only 24 h, other trials used low-dose glucocorticoids. In addition, TSA indicated that current evidence regarding new infection is inconclusive and further studies are needed.

Several limitations need to be considered in our meta-analysis. First, due to a lack of individual patient data, we were unable to conduct subgroup analyses according to patient baseline characteristics such as the underlying etiology of ARDS. Second, the study population and corticosteroid regimen varied among studies, which might result in clinical heterogeneity. More data are needed to evaluate the impact of corticosteroid regimen on outcomes. Third, two trials were judged to be at high risk of performance and detection bias in that these studies were not blinded to the study protocol, which might compromise the reliability of our results. Finally, there was a potential risk of publication bias in our study. It has been frequently noted that small trials tend to yield more extreme effects than large trials and are particularly susceptible to publication bias. After excluding small studies with low weight, our results did not alter significantly, indicating that our findings were reliable.

## Conclusions

Our meta-analysis indicated that glucocorticoid treatment might reduce overall mortality and duration of mechanical ventilation and improve oxygenation in patients with ARDS. Glucocorticoids might be recommended as an adjunct to standard care for ARDS; however, the optimal dose and duration of steroid therapy remains unknown and further studies are needed.

## Supplementary Information

**Additional file 1.** Search strategy terms.

**Additional file 2.** Test for publication bias for hospital mortality. *RR* relative risk.

**Additional file 3.** Trial sequential analysis of eight trials for new infection. The required information size for detecting an intervention effect was 2131 patients. The relative risk was 0.84, and the 95% confidence interval was corrected to 0.66–1.08, from 0.70 to 1.01. TSA showed that the cumulative *Z*-curve did not cross both the conventional boundary and the trial sequential monitoring.

**Additional file 4.** Trial sequential analysis of hyperglycemia trials for hyperglycemia. The required information size for detecting an intervention effect was 746 patients. The relative risk was 1.11, and the 95% confidence interval was corrected to 0.98–1.25, from 0.99 to1.23. TSA showed that the cumulative *Z*-curve crossed the futility boundary.

## Data Availability

Not applicable.

## Abbreviations

ARDS: Acute respiratory distress syndrome
ICU: Intensive care unit
RCT: Randomized controlled trial
RR: Risk ratio
CI: Confidence interval
MD: Mean difference
SMD: Standardized mean difference
SD: Standard deviation
TSA: Trial sequential analysis
HPA: Hypothalamic–pituitary–adrenal
CIRCI: Critical illness-related corticosteroid insufficiency
