# Supplementary material for: Decreased mortality in acute respiratory distress syndrome patients treated with corticosteroids: an updated meta-analysis of randomized clinical trials with trial sequential analysis
Source: Crit Care. 2021 Mar 26;25:122. doi: 10.1186/s13054-021-03546-0 (PMC7995395; doi:10.1186/s13054-021-03546-0)
Supplement: Supplementary file 1 — Additional file 1. Search strategy terms. [file 13054_2021_3546_MOESM1_ESM.doc]

**Additional file 1** Search strategy terms

#1 acute respiratory distress syndrome.mp. [mp=ti, ot, ab, sh, hw, kw, tn, dm, mf, dv, fx, dq, nm, kf, ox, px, rx, an, ui, sy]

#2 ARDS.ti,ab.

#3 acute lung injury.ti,ab.

#4 ALI.ti,ab.

#5 1 or 2 or 3 or 4

#6 glucocorticoids.mp. [mp=ti, ot, ab, sh, hw, kw, tn, dm, mf, dv, fx, dq, nm, kf, ox, px, rx, an, ui, sy]

#7 corticosteroid.ti,ab.

#8 corticoid.ti,ab.

#9 steroids.ti,ab

#10 hydrocortisone.ti,ab.

#11 prednisolone.ti,ab.

#12 dexamethasone.ti,ab.

#13 methylprednisolone.ti,ab.

#14 6 or 7 or 8 or 9 or 10 or 11 or 12 or 13

#15 randomized controlled trial.mp. [mp=ti, ot, ab, sh, hw, kw, tn, dm, mf, dv, fx, dq, nm, kf, ox, px, rx, an, ui, sy]

#16 controlled clinical trial.ti,ab.

#17 randomized.ti,ab.

#18 randomly.ti,ab.

#19 trial.ti,ab.

#20 15 or 16 or 17 or 18 or 19

#21 5 and 14 and 20

#22 limit 21 to human
