# Supplementary figures and images for: Decreased mortality in acute respiratory distress syndrome patients treated with corticosteroids: an updated meta-analysis of randomized clinical trials with trial sequential analysis
Source: Crit Care. 2021 Mar 26;25:122. doi: 10.1186/s13054-021-03546-0 (PMC7995395; doi:10.1186/s13054-021-03546-0)

**Additional file 2** Test for publication bias for hospital mortality. RR, relative risk.

**
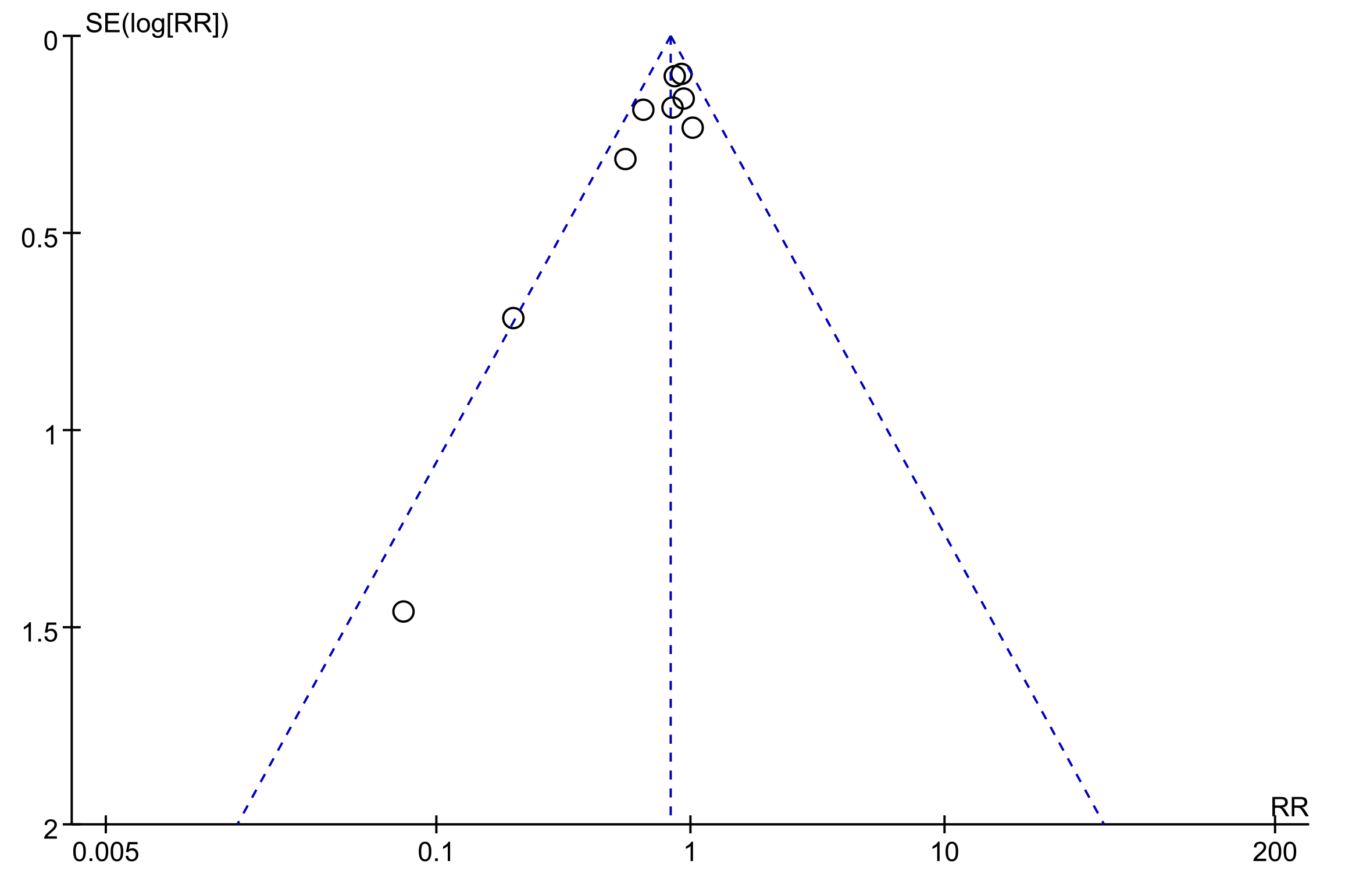
**

Supplement: Supplementary file 2 — Additional file 2. Test for publication bias for hospital mortality. RR relative risk. [file 13054_2021_3546_MOESM2_ESM.doc]
