# Supplementary material for: Decreased mortality in acute respiratory distress syndrome patients treated with corticosteroids: an updated meta-analysis of randomized clinical trials with trial sequential analysis
Source: Crit Care. 2021 Mar 26;25:122. doi: 10.1186/s13054-021-03546-0 (PMC7995395; doi:10.1186/s13054-021-03546-0)

**Additional file 3** Trial sequential analysis of eight trials for new infection. The required information size for detecting an intervention effect was 2131 patients. The relative risk was 0.84, and the 95% confidence interval was corrected to 0.66 to 1.08, from 0.70 to 1.01. TSA showed that the cumulative Z-curve did not crosse both the conventional boundary and the trial sequential monitoring boundary.


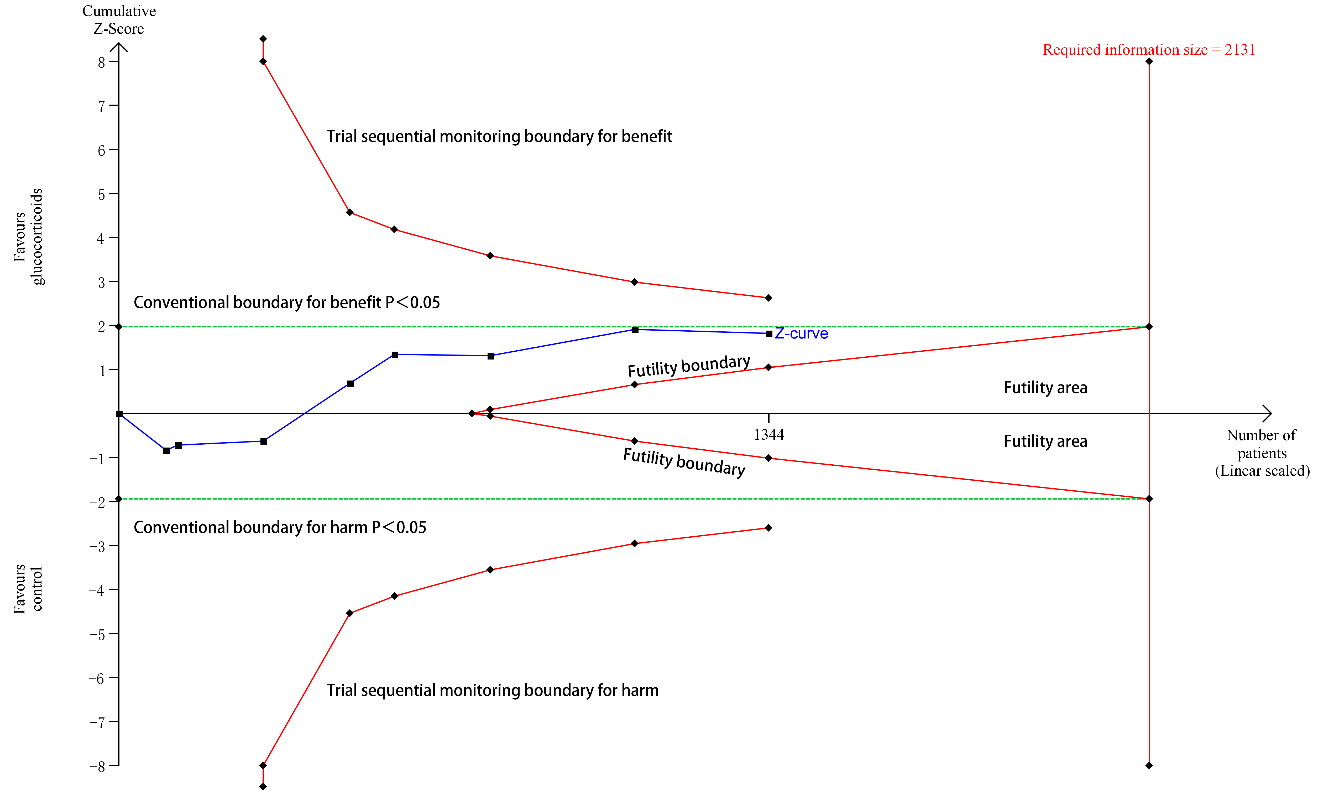

Supplement: Supplementary file 3 — Additional file 3. Trial sequential analysis of eight trials for new infection. The required information size for detecting an intervention effect was 2131 patients. The relative risk was 0.84, and the 95% confidence interval was corrected to 0.66–1.08, from 0.70 to 1.01. TSA showed that the cumulative Z-curve did not cross both the conventional boundary and the trial sequential monitoring. [file 13054_2021_3546_MOESM3_ESM.doc]
